# Physics-informed extreme learning machine (PIELM) for consolidation around an expanded cylindrical cavity

**DOI:** 10.1371/journal.pone.0329789

**Published:** 2025-08-14

**Authors:** Chuan-Qin Pang, Zhu-Hao Zhang, Si-Han Chen, Hong-Ya Yue, Yu Zhang

**Affiliations:** 1 Shandong Provincial Key Laboratory of Intelligent Construction and Maintenance of Highway Infrastructure, Shandong Jiaotong University, Jinan, China; 2 Shandong Urban Construction Vocational College, Jinan, China; 3 Shandong Provincial Communications Planning and Design Institute Group Co., Ltd, Jinan, China; 4 School of Qilu Transportation, Shandong University, Jinan, China; China University of Mining and Technology, CHINA

## Abstract

This paper proposes a physics-informed extreme learning machine (PIELM) for analyzing consolidation immediately after cavity expansion. The deep neural networks in traditional physics-informed neural network (PINN) framework are substituted by the extreme learning machine (ELM) network with only one hidden layer. By using exact definition of stress invarients, the distribution of excess water pressure after cavity expansion is rigorously incorporated into PIELM framework as initial conditions. Then, a loss vector is obtained by combining governing equation, initial conditions and boundary conditions, and the ELM network can be directly trained by optimising the loss vector via the least squares method. It is found that: (i) the PIELM approach can provide accurate prediction for consolidation analysis after cavity expansion; and (ii) the dissipation of excess water pressure heavily relies on its initial distribution that is related to soil mechanical behaviour. This proposed approach can serve as an efficient tool to interpret consolidation coefficient from piezocone penetration tests (CPTU) with measured data.

## 1. Introduction

Consolidation of saturated soils refers to the time-dependent process in which the excess water pressure dissipates and soil volume decreases. Since Terzaghi [1] proposed the first theory for soil consolidation, the consolidation theory has played a critical role in geotechnical engineering in predicting the long-term ground settlement [2–4]. In particular, soil consolidation around an expanded cavity is significant for assessing the time-dependent bearing capacity of driven piles [5–9] and interpreting piezocone cone penetration tests [10–12].

Since the work of Randolph and Wroth [13], various studies have been published to quantify the consolidation process after an expanded cavity. In Randolph and Wroth [13], the soil plasticity is modeled by a perfectly plastic model that satisfies Tresca yield criterion. Zhou, et al. [14] extended the work of Randolph and Wroth [13] by using the modified Cam Clay model to simulate more sophisticated soil behaviour, where the time-dependent excess water pressure was calculated by the finite difference method. Li, et al. [7] presented an analytical solution for consolidation after cavity expansion to quantify the time-dependent bearing capacity of driven piles. In these studies, however, the solution for initial water pressure distribution is approximate because the definitions of stress invariants are not rigorous [15,16]. When using Cam Clay models, to the best knowledge of the authors, exact solution for consolidation after cavity expansion has not been available so far.

Recent development of physics-informed machine learning (PIML) enables ordinary or partial differential equations (ODEs/PDEs) to be solved by combining neural networks and physical laws [17–21]. Compared to the finite element method and finite difference method, it is a mesh-free method that makes use of the universal function approximation capability of neural networks. As one of the most important branches of PIML, physics-informed neural networks (PINNs) solve ODEs/PDEs with the aid of deep neural networks, and have been widely applied in geotechnical engineering problems, such as cavity expansion [22,23], consolidation [24–28], and soil-structure interaction [27,29]. However, conventional PINNs normally require high computation time for training neural networks, and the network hyperparameters are not easily determined. To overcome this limitation, physics-informed extreme learning machine (PIELM) proposed by Dwivedi and Srinivasan [30] is another powerful PIML technique, which replaces the deep neural network in the PINN framework by a single hidden-layer extreme learning machine (ELM) [31]. Compared to conventional PINNs, PIELM can solve various linear or nonlinear ODEs/PDEs with high efficiency and accuracy [30,32–36]. For example, Ren, et al. [35] solved the Stefan problems by PIELM, which saves more than 90% training time and improves the solution accuracy from 10^−3^ ~ 10^−5^ to 10^−6^ ~ 10^−8^. However, the application of PIELM to consolidation analysis has rarely been reported.

Given the high performance of PIELM, this paper applies PIELM for consolidation analysis after an expanded cavity. First, the distribution of excess water pressure immediately after cavity expansion is obtained based on the cavity expansion theory, and rigorous definitions of stress invariants are adopted in the cavity expansion stage. Then, the PIELM framework for solving equations is presented, incorporating normalised consolidation equation, initial conditions and boundary conditions. Later, the PIELM approach for consolidation is validated and parametric studies are conducted. Finally, main conclusions are drawn.

## 2. Problem definition

**Fig 1:**
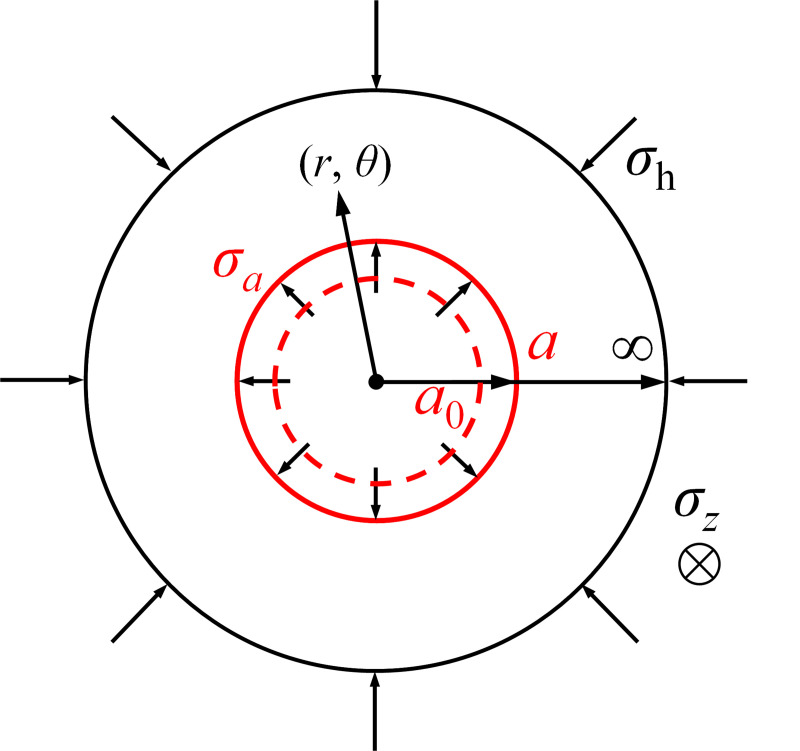
Schematic of cavity expansion.

Initially, it is assumed that a cylindrical cavity is embedded in the isotropic, homogeneous and fully saturated soil, as shown in Fig 1. The inner cavity radius is denoted as *a*_0_ and the outer boundary has an infinite radial extent. The constitutive behaviour is modelled by the modified Cam Clay (MCC) model, and water flow is assumed to obey Darcy’s law. The soil is subjected to horizontal and vertical stresses ($\sigma_{h}$, $\sigma_{v}$). Then the cavity pressure gradually increases to $\sigma_{a}$, leading to the cavity expanding outwards. At the same time, a plastic zone form, characterised by an elastoplastic radius *ρ*. When the inner cavity radius reaches *a*, the expansion process ends and the consolidation process begins immediately [6,13,14]. The plane strain assumption is satisfied and the cylindrical coordinate (*r*, *θ*, *z*) is used to account for the cavity expansion and consolidation processes.

## 3. Cavity expansion solution and distribution of excess water pressure

### 3.1. Governing equations

Before the consolidation analysis, a solution should be developed for the distribution of excess water pressure after cavity expansion. The solution method in Chen and Abousleiman [37] and Yang, et al. [38] is used in this paper as follows.

The considered cavity expansion problem can be formulated by the stress equilibrium equation, compatibility equations, and stress-strain relationship. When the dynamic effect is ignored, the stress equilibrium equation is expressed as


$$\frac{d\sigma_{r}^{\prime }}{dr}+\frac{du_{w1}}{dr}+\frac{\sigma_{r}^{\prime }-\sigma_{\theta }^{\prime }}{r}=0$$ (1)

where $\sigma_{r}^{\prime }$ and $\sigma_{\theta }^{\prime }$ are the radial and circumferential effective stresses, respectively; *u*_w1_ is pore water pressure during the cavity expansion process, and the initial ambient water pressure is *u*_w0_; *r* is the radial position of a soil material point; d()/d()dr\nulldelimiterspacedr denotes the differential in terms of *r*. In this paper, the stresses and strains are taken as positive under compression.

It is assumed that the small strain theory is satisfied in the elastic zone, as


$$\varepsilon_{r}=-\frac{du}{dr}$$ (2)

$$\varepsilon_{\theta }=-\frac{u}{r}$$ (3)

where $u=r-r_{0}$ and $r_{0}$ is the initial radial position of the soil material point; $\varepsilon_{r}$ and $\varepsilon_{\theta }$ are the radial and tangential strains, respectively. In the plastic zone, the large strain definition is chosen because large plastic deformation appears:


$$\varepsilon_{r}=-\ln\frac{dr}{dr_{0}}$$ (4)

$$\varepsilon_{\theta }=-\ln\frac{r}{r_{0}}$$ (5)

$$\varepsilon_{v}=\varepsilon_{r}+\varepsilon_{\theta }=0$$ (6)

where $\varepsilon_{v}$ is the volumetric strain and remains zero for saturated soil under undrained conditions.

The MCC model is adopted for constitutive modelling of the soil, including the yield function, plastic potential, Hooke’s law, and the critical state line:


f=(q/qMp′\nulldelimiterspaceMp′)2−[pc′/pc′p′\nulldelimiterspacep′−1]= 0 (7)

g=(q/qMp′\nulldelimiterspaceMp′)2−[pc′/pc′p′\nulldelimiterspacep′−1]= 0 (8)

$$\begin{array}{c} p^{\prime }=\frac{\sigma_{r}^{\prime }+\sigma_{\theta }^{\prime }+\sigma_{z}^{\prime }}{3} \end{array}$$ (9)

$$q=\frac{\sqrt{2}}{2}\sqrt{{\left(\sigma_{r}^{\prime }-\sigma_{\theta }^{\prime }\right)}^{2}+{\left(\sigma_{r}^{\prime }-\sigma_{z}^{\prime }\right)}^{2}+{\left(\sigma_{\theta }^{\prime }-\sigma_{z}^{\prime }\right)}^{2}}$$ (10)

$$\left[\;\;\begin{array}{c} D\varepsilon_{r}^{e}\\ D\varepsilon_{\theta }^{e}\\ D\varepsilon_{z}^{e} \end{array}\;\;\right]=\frac{1}{E}\left[\begin{array}{ccc} {}*20c1 & -\mu & -\mu \\ -\mu & 1 & -\mu \\ -\mu & -\mu & 1 \end{array}\right]\left[\;\;\begin{array}{c} {}*20cD\sigma_{r}^{\prime }\\ D\sigma_{\theta }^{\prime }\\ D\sigma_{z}^{\prime } \end{array}\;\;\right]$$ (11)

$$\begin{array}{c} E=3\left(1-2\mu \right)\frac{vp^{\prime }}{\kappa } \end{array}$$ (12)

$$\begin{array}{c} v=\Gamma -\lambda \ln p^{\prime } \end{array}$$ (13)

where *f* and *g* are the yield function and plastic potential respectively, and *f* = g for the associated flow rule; $\begin{array}{c} p^{\prime } \end{array}$ and *q* are the mean effective stress and deviatoric stress, respectively; *M* is the slope of the critical state line in the $\begin{array}{c} p^{\prime }-q \end{array}$ plane; $\begin{array}{c} p_{c}^{\prime } \end{array}$ denotes the isotropic yield stress; $\begin{array}{c} \sigma_{z}^{\prime } \end{array}$ is the vertical effective stress; D means the material time derivative; $\varepsilon_{r}^{e}$, $\varepsilon_{\theta }^{e}$ and $\varepsilon_{z}^{e}$ are the elastic radial, circumferential and vertical strains, respectively; *E* is the elastic modulus and *µ* is Poisson’s ratio; *v* is the specific volume of soil; λ and κ are the slopes of the critical state line and swelling line in the $\begin{array}{c} v-\ln p^{\prime } \end{array}$ plane.

### 3.2. Cavity expansion solution

Following Chen and Abousleiman [37] and Yang, et al. [38], the stress components, radial displacement, and pore water pressure in the elastic zone can be obtained as


$$\sigma_{r}=\sigma_{h}+\left(\sigma_{r\rho }-\sigma_{h}\right){\left(\frac{\rho }{r}\right)}^{2}$$ (14)

$$\sigma_{\theta }=\sigma_{h}-\left(\sigma_{r\rho }-\sigma_{h}\right){\left(\frac{\rho }{r}\right)}^{2}$$ (15)

$$\sigma_{z}=\sigma_{v}$$ (16)

$$\frac{u}{r}=\frac{1+\mu }{E}\left(\sigma_{r\rho }-\sigma_{h}\right){\left(\frac{\rho }{r}\right)}^{2}$$ (17)

$$u_{w1}=u_{w0}$$ (18)

where $\sigma_{r\rho }$ is the radial stress at the elastoplastic boundary. By substituting Eqs. (14), (15) and (16) into Eq. (7), the radial and circumferential effective stresses at the elastoplastic boundary (i.e., $\sigma_{r\rho }^{\prime }$ and $\sigma_{\theta \rho }^{\prime }$) can be derived as


$$\begin{array}{c} \sigma_{r\rho }^{\prime }=\sigma_{h}^{\prime }+\sqrt{\sigma_{h}^{\prime 2}-\frac{1}{3}\left(4\sigma_{h}^{\prime 2}+\sigma_{v}^{2}-2\sigma_{h}\sigma_{v}\right)-q_{\rho }^{2}} \end{array}$$ (19)

$$\begin{array}{c} \sigma_{\theta \rho }^{\prime }=\sigma_{h}^{\prime }-\sqrt{\sigma_{r}^{\prime 2}-\frac{1}{3}\left(4\sigma_{h}^{\prime 2}+\sigma_{v}^{2}-2\sigma_{h}\sigma_{v}\right)-q_{\rho }^{2}} \end{array}$$ (20)

$$q_{\rho }=\frac{1}{\sqrt{2}}\sqrt{{\left(\sigma_{r\rho }^{\prime }-\sigma_{\theta \rho }^{\prime }\right)}^{2}+{\left(\sigma_{r\rho }^{\prime }-\sigma_{h}^{\prime }\right)}^{2}+{\left(\sigma_{\theta \rho }^{\prime }-\sigma_{h}^{\prime }\right)}^{2}}$$ (21)

In the plastic zone, the incremental stress-strain relationship can be shown as


$$\left[\begin{array}{ccc} {}*20ca_{11} & a_{12} & a_{13}\\ a_{21} & a_{22} & a_{23}\\ a_{31} & a_{32} & a_{33} \end{array}\right]\left[\begin{array}{c} {}*20cD\sigma_{r}^{\prime }\\ D\sigma_{\theta }^{\prime }\\ D\sigma_{z}^{\prime } \end{array}\right]=\left[\begin{array}{c} {}*20cD\varepsilon_{\theta }\\ D\varepsilon_{z}\\ D\varepsilon_{v} \end{array}\right]$$ (22)

in which


$$a_{11}=\frac{-\mu }{E}+\frac{1}{K_{p}}\frac{\partial f}{\partial \sigma_{r}^{\prime }}\frac{\partial g}{\partial \sigma_{\theta }^{\prime }}$$ (23)

$$a_{12}=\frac{1}{E}+\frac{1}{K_{p}}\frac{\partial f}{\partial \sigma_{\theta }^{\prime }}\frac{\partial g}{\partial \sigma_{\theta }^{\prime }}$$ (24)

$$a_{13}=\frac{-\mu }{E}+\frac{1}{K_{p}}\frac{\partial f}{\partial \sigma_{z}^{\prime }}\frac{\partial g}{\partial \sigma_{\theta }^{\prime }}$$ (25)

$$a_{21}=\frac{-\mu }{E}+\frac{1}{K_{p}}\frac{\partial f}{\partial \sigma_{r}^{\prime }}\frac{\partial g}{\partial \sigma_{z}^{\prime }}$$ (26)

$$a_{22}=\frac{-\mu }{E}+\frac{1}{K_{p}}\frac{\partial f}{\partial \sigma_{\theta }^{\prime }}\frac{\partial g}{\partial \sigma_{z}^{\prime }}$$ (27)

$$a_{23}=\frac{1}{E}+\frac{1}{K_{p}}\frac{\partial f}{\partial \sigma_{z}^{\prime }}\frac{\partial g}{\partial \sigma_{z}^{\prime }}$$ (28)

$$\begin{array}{c} a_{31}=\frac{1-2\mu }{E}+\frac{1}{K_{p}}\frac{\partial f}{\partial \sigma_{r}^{\prime }}\frac{\partial g}{\partial p^{\prime }} \end{array}$$ (29)

$$\begin{array}{c} a_{32}=\frac{1-2\mu }{E}+\frac{1}{K_{p}}\frac{\partial f}{\partial \sigma_{\theta }^{\prime }}\frac{\partial g}{\partial p^{\prime }} \end{array}$$ (30)

$$\begin{array}{c} a_{33}=\frac{1-2\mu }{E}+\frac{1}{K_{p}}\frac{\partial f}{\partial \sigma_{z}^{\prime }}\frac{\partial g}{\partial p^{\prime }} \end{array}$$ (31)

$$K_{p}=\frac{vp\prime }{\lambda -\kappa }\frac{M^{2}-\eta^{2}}{M^{2}{p\prime }^{2}}$$ (32)

Substituting Eqs. (4), (5) and (6) into Eq. (22), the effective stress components can be expressed as three ordinary differential equations (ODEs) in terms of *r*:


[*20ca21a22a23a31a32a33a41a42a43][*20cDσr′Dσθ′Dσz′]=[*20c−Dr/−Drr\nulldelimiterspacer00] (33)

The ODEs can be solved numerically by giving Dr/Drr\nulldelimiterspacer, and then the pore water pressure can be obtained by integrating Eq. (1) over [*ρ*, *r*]:


$$u_{w1}=u_{w0}+\sigma_{r\rho }^{\prime }-\sigma_{r}^{\prime }+\int_{\rho }^{r}\frac{\sigma_{\theta }^{\prime }-\sigma_{r}^{\prime }}{r}dr$$ (34)

The current distribution of pore water pressure $u_{w1}$ will be set as the initial conditions for consolidation analysis after cavity expansion.

## 4. PIELM for consolidation analysis

In this section, the consolidation equation is given and normalised, and the consolidation process is analysed using PIELM.

### 4.1. Physical laws and variable normalisation

According to Randolph and Worth (1978) and Zhou, et al. [14], it is assumed that only elastic deformation occurs during the consolidation process. Then the PDE as well as the initial and boundary conditions for consolidation after cavity expansion can be written as


$$\frac{\partial u_{w2}}{\partial t}=c\left(\frac{\partial^{2}u_{w2}}{\partial r^{2}}+\frac{1}{r}\frac{\partial u_{w2}}{\partial r}\right)$$ (35)

$${u_{w2}\left(a,t\right)|}_{t=0}=u_{w1}$$ (36)

$${\frac{\partial u_{w2}}{\partial r}|}_{r=a}=0$$ (37)

$$u_{w2}\left(b,t\right)=u_{w0}$$ (38)

where *t* is the consolidation time and *t* = 0 means the end of cavity expansion (also the start of consolidation); $u_{w2}$ is the pore water pressure during the dissipation process; *c* is the consolidation coefficient and is assumed as a constant in this paper; *b* is a sufficiently large value representing the infinite boundary.

To conveniently solve the consolidation equation by PIELM, the PDE is normalised with


$$U=\frac{u_{w2}-u_{w0}}{u_{w}^{ref}}$$ (39)

$$T=\frac{ct}{a^{2}}$$ (40)

$$R=\frac{r}{a}$$ (41)

where $u_{w}^{ref}={\left(u_{w2}-u_{w0}\right)|}_{r=a}$ is the reference pore water pressure. Then the PDE, initial conditions and boundary conditions


$$\frac{\partial U}{\partial T}=\frac{\partial^{2}U}{\partial R^{2}}+\frac{1}{R}\frac{\partial U}{\partial R}$$ (42)

$${U\left(1,T\right)|}_{t=0}=\frac{u_{w1}-u_{w0}}{u_{w}^{ref}}=U_{0}\left(R\right)$$ (43)

$${\frac{\partial U\left(1,T\right)}{\partial R}|}_{R=1}=0$$ (44)

U(b/ba\nulldelimiterspacea,T)=0 (45)

### 4.2. PIELM framework

**Fig 2:**
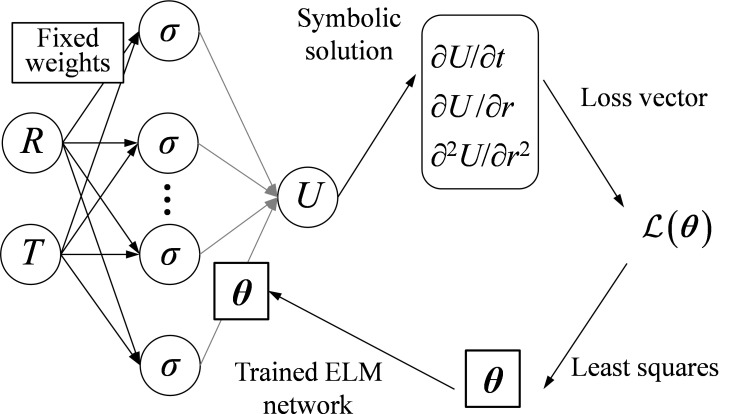
PIELM framework for consolidation analysis.


As aforementioned, conventional PINN solves PDEs with the aid of deep neural networks, but the computation cost for training neural networks is heavy. To accelerate the training process, the deep neural network is replaced with the ELM, and the PIELM framework is shown in Fig 2. In the ELM there is only a single hidden layer with a few neurons. First, *N*_c_ collocation data ${\left\{R_{i},T_{i}\right\}}_{i=1}^{N_{c}}$ are generated, where $R_{i}\in \left[1,\;b/a\right]$ and $T_{i}\in \left[0,T_{\max}\right]$. These collocation data are set as inputs and fed into the ELM network, whose input layer weights are randomly generated in the range of [−1,1]. The inputs are transformed after passing through a single hidden layer fully connected neural network which randomly assigns input layer weights (will not be trained). Then, the normalised pore water pressure as well as their derivatives can be shown in the symbolic expression. They are incorporated into the loss vectors with physical laws:

$$L=\left[L_{pde}^{\left(1\right)}\left(\theta \right),...,L_{pde}^{\left(N_{c}\right)}\left(\theta \right),L_{bc1}^{\left(1\right)}\left(\theta \right),...,L_{bc1}^{\left(N_{B}\right)}\left(\theta \right),L_{bc2}^{\left(1\right)}\left(\theta \right),...,L_{bc2}^{\left(N_{B}\right)}\left(\theta \right),L_{ic}^{\left(1\right)}\left(\theta \right),...,L_{ic}^{\left(N_{I}\right)}\left(\theta \right)\right]\to 0$$ (46)

$$L_{pde}^{\left(i\right)}\left(\theta \right)=\frac{\partial U\left(R_{i},T_{i};\theta \right)}{\partial T}-\left(\frac{\partial^{2}U\left(R_{i},T_{i};\theta \right)}{\partial {R_{i}}^{2}}+\frac{1}{R_{i}}\frac{\partial U\left(R_{i},T_{i};\theta \right)}{\partial R_{i}}\right)\;i=1,2,...,N_{c}$$ (47)

$$L_{bc1}^{\left(j\right)}\left(\theta \right)=\frac{\partial U\left(1,T_{j}\right)}{\partial R}\;j=1,2,...,N_{B}$$ (48)

Lbc2(j)(θ)=U(b/ba\nulldelimiterspacea,Tj)j=1,2,...,NB (49)

$$L_{ic}^{\left(l\right)}\left(\theta \right)=U\left(R_{l},0;\theta \right)-U_{0}\left(R_{l}\right)\;l=1,2,...,N_{I}$$ (50)

where ${\left\{R_{l},0\right\}}_{l=1}^{N_{I}}$, ${\left\{1,T_{j}\right\}}_{j=1}^{N_{B}}$ ${\left\{b/a,T_{j}\right\}}_{j=1}^{N_{B}}$ are points for initial and boundary conditions, in which $R_{l}\in \left[1,\;b/a\right]$, $T_{j}\in \left[0,T_{\max}\right]$. ***θ*** denotes the output layer parameters in ELM networks, and it can be directly solved by the least squares method with the Moore–Penrose generalised inverse. Instead, PINNs obtain the layer weights by the time-consuming gradient descent method, thereby greatly increasing the training time.

## 5. Results and discussion

### 5.1. Validation

**Fig 3:**
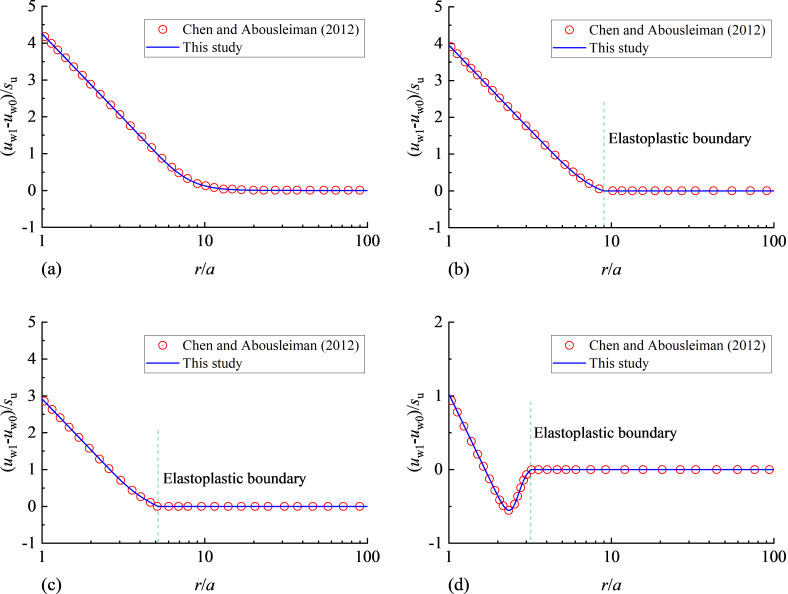
Distribution of water pressure immediately after cavity expansion: (a) *R*_0_ = 1; (b) *R*_0_ = 1.2; (c) *R*_0_ = 3; (d) *R*_0_ = 10.


The water pressure distribution at the end of expansion stage is compared with the exact solution of Chen and Abousleiman [37], as shown in Fig 3. The input parameters are the same as Chen and Abousleiman [37] and are summarised in Table 1. The excess water pressure is normalised by the undrained shear strength *s*_u_:

**Table 1:** Input parameters in Chen and Abousleiman [37].

*R*_0_	$\sigma \prime_{h}$ (kPa)	$\sigma \prime_{v}$ (kPa)	*u*_w0_ (kPa)	Material parameters
1	100	160	100	*M* = 1.2 *λ* = 0.15 *κ* = 0.03 *μ* = 0.278 *Γ* = 2.74
1.2	100	160	100	
3	120	120	100	
10	144	72	100	

Note: *R*_0_ is the overconsolidation ratio.

$$s_{u}=\frac{1}{\sqrt{3}}M\exp\left(\frac{\Gamma -v}{\lambda }\right)$$ (51)

It can be found from Fig 3 that the distributions of excess water pressure in this paper match well with the exact solution of Chen and Abousleiman [37], which will be incorporated into the PIELM framework as the initial conditions.

Furthermore, the accuracy of PIELM in the consolidation stage is validated by comparison with Randolph and Wroth [13]’s analytical solution. When the soil is modeled by the Tresca model, the distribution of water pressure at the end of cavity expansion can be expressed as


uw1=2suln(ρ/ρr\nulldelimiterspacer) (52)

**Fig 4:**
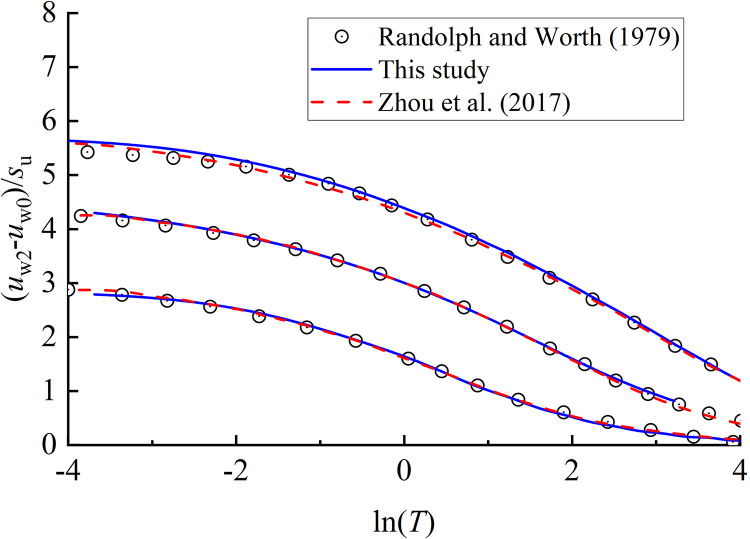
Comparison of excess water pressure predicted by PIELM, analytical solution and finite difference method.

ρ/ρa\nulldelimiterspacea=G/Gsu\nulldelimiterspacesu (53)

where *G* is the shear modulus of soil. With this distribution of water pressure, the evolution of water pressure during the subsequent consolidation process is shown in Fig 4, which are derived by PIELM and analytical solution, respectively. For PIELM method, *N*_*c*_ = 40000, *N*_*I*_ = 100, *N*_*B*_ = 1000, *b* = 100, and 1000 neurons and activation function tanh are selected in the hidden layer of ELM network. Using the Matlab R2023b on a Dell computer with Intel(R) Core(TM) i7-11700 @ 2.50GHz processor and 16GB of RAM memory, it takes 10–20 seconds to train the ELM network, which is much more efficient than conventional PINN. Generally, the excess water pressure predicted by PIELM is in good agreement with the Randolph and Wroth [13]’s analytical solution and Zhou, et al. [14]’s finite difference solution. Therefore, the soundness of PIELM has been validated.

### 5.2. Dissipation of excess water pressure

**Fig 5:**
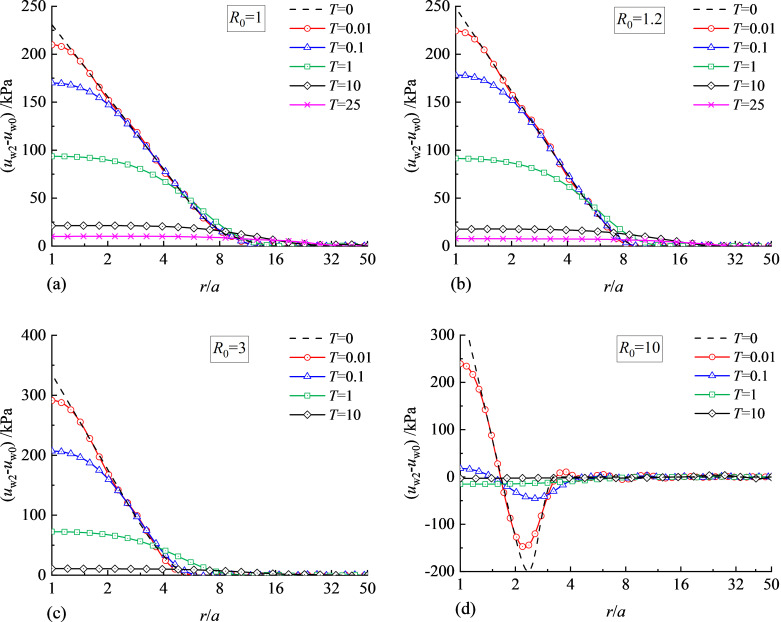
Distribution of excess water pressure with various overconsolidation ratio: (a) *R*_0_ = 1; (b) *R*_0_ = 1.2; (c) *R*_0_ = 3; (d) *R*_0_ = 10.

Fig 5 shows the distribution of excess water pressure with various overconsolidation ratios. The input parameters for the cavity expansion process are the same as listed in Table 1. The results for *a*/*a*_0_ = 2 and *b* = 100 are selected, and the training points for PIELM are set as *N*_*c*_ = 40000, *N*_*I*_ = 200, *N*_*B*_ = 200. 1000 neurons are used in the hidden layer and the hyperbolic tangent function (tanh) is chosen as the activation function. It shows that the excess water pressure is positive when *R*_0_ ≤ 3, while negative excess water pressure occurs for highly overconsolidated soils (*R*_0_ = 10). Compared to the initial distribution of excess water pressure (i.e., *T* = 0), the excess water pressure at the inner cavity wall rapidly drops on the occasion of *T* = 0.01 because of a higher water pressure gradient. With the increase of consolidation time, the excess water pressure gradually approaches zero and the consolidation process ends after *T* = 25. Besides, the dissipation of excess water pressure depends on *R*_0_, which determines the initial distribution of excess water pressure. In other words, the interpretation of consolidation coefficient from piezocone penetration tests (CPTU) should take mechanical parameters of soils into consideration.

Finally, the effect of definitions of stress invariants is investigated. As shown in Collins and Yu (1996), the stress invariants during the cavity expansion process are simplified as


$$p\prime =\frac{\sigma_{r}^{\prime }+\sigma_{\theta }^{\prime }}{2}$$ (54)

$$q=\frac{\sigma_{r}^{\prime }-\sigma_{\theta }^{\prime }}{2}$$ (55)

**Fig 6:**
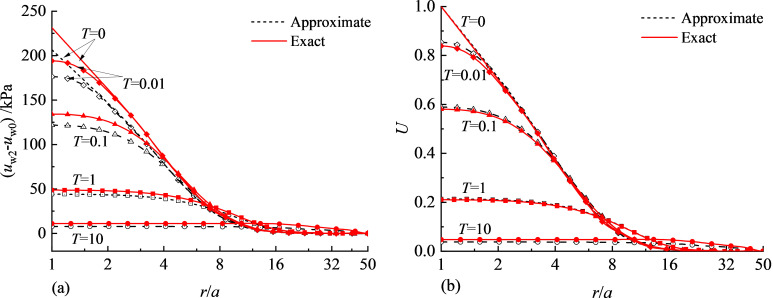
Comparison of pore water pressure between exact and approximate definitions of stress invariants: (a) Excess water pressure; (b) Normalised water pressure.

In this case the distribution of excess water pressure immediately after cavity expansion will be different from that using rigorous definitions of p′ and *q* in Eqs. (9) and (10). Fig 6 shows the comparison of pore water pressure between exact and approximate stress invariant definitions. The input parameters for cavity expansion are: *M* = 0.773, *λ* = 0.161, *κ* = 0.062, *μ* = 0.3, *Γ* = 2.759, *v* = 2.0, $\sigma_{h}$=$\sigma_{v}$=170.8kPa. It shows that the excess water pressure is underestimated when the approximate definitions of stress invariants are adopted, as shown in Fig 6(a). However, when the excess water pressure is normalised as *U* using Eq. (39), the distributions of *U* with the two definitions are nearly identical. This means that the distribution patterns for excess water pressure are similar regarding rigorous and approximate definitions of stress invariants.

## Conclusions

This paper proposes a physics-informed extreme learning machine (PIELM) method for consolidation analysis after the cavity expansion process. Rigorous solution for initial pore water pressure distribution is adopted by taking exact definitions of stress invariants for cavity expansion analysis. Then, the solution provides initial conditions incorporated into extreme learning machine (ELM) network, and a loss vector is formulated by combining the consolidation equation, initial conditions, and boundary conditions. Different from the conventional physics-informed neural network (PINN), PIELM obtains the network parameters directly by minimising the loss vector via the least squares method. The accuracy of PIELM is validated by comparison with published analytical solution and finite difference method. Finally, parametric studies are conducted, showing the significance of the overconsolidation ratio on the dissipation of excess water pressure after cavity expansion.

## Supporting information

S1 File Data for figures 3–5. (ZIP)
